# Annotations

**Published:** 1897-04-10

**Authors:** 


### Annotations.

### The Nomenclature of Diseases.

Those who have amused themselves with a study of the "Nomenclature of Diseases," published under the auspices of the Royal College of Physicians, a third edition of which was issued only last year, will not be surprised to find that questions have arisen as to the meaning of the synonyms interspersed throughout that work. The Local Government Board whether, having regard to the fact that in the third edition of the "Nomenclature of Diseases" "membranous croup" is stated to be a synonym for "laryngeal diphtheria," they consider that patients certified as suffering from "membranous croup" without qualification may properly be admitted into their hospitals. In answer to this the Local Government Board state that they are advised that the term "membranous croup" *per se* is not sufficiently definite, inasmuch as membranous inflammation of the larynx may arise from causes other than diphtheria. With this decision, of course, everyone will agree. It will, however, be likely to make medical men all the more dissatisfied with the nomenclature which they are asked to use in the registration of disease. Let us look up this word "croup" in the index. It is not to be found! There is "*Croup, spasmodic*, see Laryngismus stridulus," indicating that spasmodic croup is to be entered under laryngismus, but there is no hint about there being any other form of disease called croup even as a synonym. In another part of the index, however, we find "*membranous croup*, see Diphtheria," and among the "Explanations" we find it stated that "the *italic* names are not recognised. To the names in *italics* are added references showing under what recognised names the disease supposed to be indicated should be returned." If that means anything it means that cases of "membranous croup" are to be returned as cases of "diphtheria," and if membranous croup is not the same as diphtheria the nomenclature clearly sanctions a false return. Is there, then, no such disease as croup? According to the index, certainly not. But hidden away on page 107 under diseases of the larynx we find it placed as a variety of membranous inflammation; in this case not in italics but in full-sized Roman type. Why is this not indexed? Even under membranous croup in the index there is no reference to this page. What are we to think of the authors of such a muddle?

### The Enlarged Functions of the Asylums Board.

The determination announced by Mr. Chaplin to hand over to the Metropolitan Asylums Board the care of those diseased and defective children who are at present in the charge of the Poor Law authorities opens up a somewhat alarming prospect to the metropolitan ratepayer. As it stands the proposal is a sufficiently simple one, but its tendencies must be judged of by a consideration of the history of the Metropolitan Asylums Board. Even in regard to its present duties this Board was at the beginning a mere Poor Law institution. But it was soon found to be impossible to refuse to the general public the advantages which the Board was able to offer to paupers in the way of hospital treatment, and from this has sprung up the great network of ambulance stations and fever hospitals by which the whole metropolis is now served. Suppose, then, the duty of treating and educating pauper children who are suffering from diseases of the eye, of the skin, of the scalp, and who are deficient in other ways, is taken up by the Asylums Board, and suppose this duty is well performed by them, as it no doubt will be, are we to imagine that the general public will not claim to have a share in the advantages of such an organisation? Education is now not only free but is compulsory. Yet it is attended with certain risks, among which is the risk of catching ringworm, a disease which is sometimes very difficult to cure, and if not cured interferes greatly with a child's prospects in life; and when it is seen that special schools are established for the reception and treatment of pauper children affected by this disease it is hardly likely that ratepaying parents will refrain from asking for their own children, when so affected, to be given the same advantages. What happened in regard to fever may well happen in regard to other disabling conditions, and it is not difficult to foresee that the Asylums Board, a body which is ever ready for work and is but little hampered by restrictions in regard to expenditure, will be led sooner or later to throw open to all the diseased and defective children of the metropolis such institutions as may be founded for the use of paupers. From an educational and sanitary point of view no doubt this would be most proper. How the ratepayers will regard it is another matter, with which of course we have nothing to do.

### The Making of Madmen.

We have again and again called attention to the evils which result from the premature discharge of the inmates of lunatic asylums, and to the fact that, gratifying as it may be to managing committees to find mention made in their reports of the large proportion of their patients who have been "cured," the result of such cures is not only an increase in the number of wandering lunatics, who, as we know from recent and sad experience, are a constant danger to the community, but also a great and continuous increase in the number of those born with a tendency to insanity. An interesting little history is told in regard to a little boy of seven years of age, who was lately returned for trial to the assizes for the murder of his brother aged six months. The mother of these children was confined in an asylum two years ago, at which time her mother and her son were also inmates; so, that at the same time, three generations of this family were officially recognized as being insane. She had several children all of them weak-minded. She was discharged "not recovered" at the instance of her husband at the end of 1895, and the child murdered was begotten and born since that time. Nothing could show more forcibly how it is that so many asylums are required. Nor is the evil resulting from this propagation of the unfit to be measured by the number of lunatics who are thereby produced. Outside and beyond what can be called lunacy there is among the offspring of the mentally feeble a large range of instability of mind, impulsiveness, inability to resist temptation, laxity of moral sense—all of which leads to crime and fills our jails and ultimately our workhouses. And yet, "at the instance of her husband," a woman can be discharged "not recovered" that she may go on adding to the multitude of the mentally unfit!

